# Implant Site Changes in Three Different Clinical Approaches: Orthodontic Extrusion, Regenerative Surgery and Spontaneous Healing after Extraction: A Systematic Review

**DOI:** 10.3390/jcm11216347

**Published:** 2022-10-27

**Authors:** Gaetano Isola, Riccardo Nucera, Silvia Damonte, Alessandro Ugolini, Anna De Mari, Marco Migliorati

**Affiliations:** 1 Department of General Surgery and Surgical-Medical Specialties, School of Dentistry, University of Catania, 95123 Catania, Italy; 2 Department of Biomedical and Dental Sciences and Morphofunctional Imaging, Section of Orthodontics, University of Messina, 98100 Messina, Italy; 3 Department of Orthodontics, Genova University, 16100 Genova, Italy

**Keywords:** clinical quality, implant site development, orthodontic extrusion, regenerative surgery, spontaneous healing, dental materials, extraction socket

## Abstract

Both surgical and non-surgical techniques are employed for implant site development. However, the efficacy of these methods has not been thoroughly evaluated and compared. This systematic review aims to compare the biologic, functional and esthetic outcomes of three different approaches before implant placement in both the maxillary and mandibular arches: orthodontic extrusion, regenerative surgery and spontaneous healing after extraction. The systematic research of articles was conducted up to January 2020 in Medline, Scopus and the Cochrane Library databases. Studies were selected in a three-stage process according to the title, the abstract and the inclusion criteria. The methodological quality and the risk of bias of the included studies were evaluated using ROBINS-I tools for non-randomized studies, Rob 2.0 for RCT. Quality evaluation of case reports was performed using CARE guidelines. Through the digital search, 1607 articles were identified, and 25 of them were included in the systematic review. The qualitative evaluation showed a good methodological quality for RCT, sufficient for non-randomized studies and poor for case reports. Based on the available results, both orthodontic extrusion and regenerative surgery allowed the development of the implant site with satisfying esthetic and functional outcomes. Studies about the spontaneous healing of the extraction socket showed resorption of the edentulous ridge, which complicated the implant insertion. No study referred to failures or severe complications. Most of the studies reported only qualitative results. The present systematic review demonstrated that there is a substantial lack of data and evidence to determine which of the presented methods is better for developing a future implant site. Both surgical and non-surgical procedures appear effective in the regeneration of hard tissue, whereas not all the techniques can improve soft tissue volume, too. The orthodontic technique simultaneously enhances both hard and soft tissue.

## 1. Introduction

The replacement of missing teeth using implants has become a routine practice in dentistry; the quality and quantity of alveolar bone and soft tissue in the future implant site are essential for proper tridimensional implant placement to achieve both esthetic and functional outcomes. 

Because of the progressive vertical and horizontal resorption of the alveolar bone following tooth loss, it is often necessary to perform some pre, peri or post-implant tissue management to ensure a good esthetic, functional and predictable long-term result for the prosthetic rehabilitation [1,2].

Several surgical techniques have been employed to develop the implant site, including bone and soft tissue grafting, guided bone regeneration (GBR), distraction osteogenesis and ridge splitting [3,4,5,6,7,8,9,10,11,12,13,14,15].

For large defects, autogenous bone blocks remain the ‘gold standard’. Ideally, the autograft is taken from an intraoral site, either the ramus or symphysis. Selection depends on the defect morphology, location of the recipient site, available bone and the position of vital structures. The ramus is often preferable; however, the presence of third molars, lack of width between the lingual wall and the external oblique ridge, or reduced bone height above the inferior dental canal may prohibit its use [16,17,18].

Symphyseal donor sites are selected according to bone availability between the inferior border of the mandible and the apices of the lower incisors and canines. A minimum safe distance of 5 mm should be left to avoid injury to these teeth. If the amount of bone needed exceeds what is available intraorally, then extraoral sites can be considered, such as the iliac crest and calvarium [16].

Autogenous bone is the ideal biomaterial. As it is a genetic match, there are no issues with biocompatibility, carcinogenicity, disease transmission, or religious or ethical objections. Compared with the other graft materials, it is more resistant to infections and has a shorter healing time. However, its single most important and unique property is its osteogenicity [16]. While other graft materials merely act as a scaffold, into which the host bone eventually grows and substitutes, an autograft will stimulate bone apposition.

A complicating factor in the use of autologous grafts is the need for a donor site with its associated morbidity. As an alternative to this type of grafting, allogenic, xenographic and alloplastic materials have been developed. However, none guarantees the healing and bone replacement that autogenous block grafts can provide [18].

Techniques such as guided bone regeneration (GBR), ridge expansion and distraction osteogenesis have the advantage of removing the need for a donor site with its associated morbidity.

Guided bone regeneration (GBR) with particulate deproteinized bovine bone mineral (DBBM) and covered with resorbable collagen membranes is currently the best documented and most widely used method to augment peri-implant alveolar bone defects [19,20]. The major drawbacks of particulate bone grafting material and collagen membrane are their poor mechanical properties, which is a key issue in hard tissue regeneration, and their low resistance to tissue collapse. Limited evidence is available regarding the hard tissue volume stability of sites that are augmented by GBR, especially at the buccal–coronal region in the anterior maxilla area, where the tissue contour is esthetically significant.

The ridge-splitting technique can increase the width of bone by splitting and expanding the existing residual ridge. Indications of this technique include the ridge deficient in width with an adequate amount of height, with no vertical defects [21]. The maxilla is preferred over the mandible because bone expansion is easily achieved without complications, thanks to the bone’s cancellous nature. Advantages include no second surgery required to place implants, reduced overall treatment time and increased implant stability because of new bone formation between cortical plates. Disadvantages include more severe ridge defects in the case of procedure failure and primary closure being difficult to achieve.

Distraction osteogenesis is based on the principle of secondary wound healing. Osteotomy removes a bone segment from the basal bone, filling the distraction gap with a callus forming a new bone segment. This procedure demonstrates many advantages, such as decreased bone resorption, no donor site morbidity and simultaneous lengthening of the surrounding soft tissues. Therefore, there are also disadvantages, such as the risk of fracture of the mandible, fracture of the transport segment, difficulties in finishing the osteotomy on the lingual side, incorrect direction of distraction, perforation of the mucosa by the transport segment, suture dehiscence, bone formation defects and pain during the activation of the distraction device [22,23,24].

To correct gingival deficiencies at potential implant sites, conventional mucogingival surgical procedures, such as connective tissue grafts, free gingival grafts and coronally positioned flaps, are the most commonly used treatment modalities [3].

Currently, orthodontic extrusion, or forced eruption, is the only non-surgical procedure for pre-implant tissue management. This is a non-invasive technique based on the periodontum ability to respond to tooth movement. Salama and Salama, in 1993, suggested using orthodontic extrusion to create vertical bone and soft tissue at hopeless tooth sites before extraction and implant placement, without the use of surgery [4].

A basic tenet of osteophysiology is that the fibers of the periodontal membrane are secured to the bone by the formation of new bone around the ends of the fibers. Bone is in a constant state of transition. As elsewhere in the body, the bone of the alveolus is constantly being resorbed and rebuilt. Therefore, when tension is applied to the periodontal ligament, periodontal fibers bundles are elongated, and osteoblasts are induced to deposit new bone in the areas where periodontal attachment exists.

The same effects on alveolar bone height are seen when orthodontic treatment is carried out with controlled and low forces which do not tear apart the periodontal fibers. In other words, a tooth moved into the dental arch by controlled extrusive orthodontic forces will bring alveolar bone, and soft tissues will follow, too [5,6].

Orthodontic extrusion is an excellent way of using teeth that are hopeless but not useless: the advantage that these teeth offer resides in their remaining attachment apparatus. This orthodontic technique manipulates the remaining attachment to augment the gingival and osseous tissues in a vertical direction. The most important benefits are the creation of a greater volume of available bone to engage the future implant and the enhancement of soft tissue volume to achieve esthetic outcomes.

Both surgical and non-surgical techniques have been employed for implant site development. However, the efficacy of these methods has not been thoroughly evaluated and compared. Therefore, deciding which approach to use is sometimes difficult and often based on personal preference instead of scientific evidence.

The aim of this systematic review is to compare the biological, functional and esthetic outcomes of orthodontic extrusion and regenerative surgery prior to implant placement in both maxillary and mandibular arch, having the case of no type of treatment of the edentulous ridge before a delayed implant placement as a control group and point of reference. The authors have identified and compared studies that examined these three different approaches.

## 2. Materials and Methods

### 2.1. Search Strategy and Study Selection

The systematic research of articles was conducted up to January 2020. The digital research was performed in the following databases: Medline, Scopus and the Cochrane Library. The following limits were activated for the search: Human, English. The search strategy was assembled with a combination of Medical Subjects Headings (MESH terms) and free-text words, as reported below:

((“orthodontic extrusion” [Mesh] OR “orthodontic extraction” OR “orthodontic eruption” OR “forced eruption” OR “OISD” OR “OFE” OR “bone graft” OR “GBR” OR “tissue regeneration” OR “bone regeneration” OR “distraction”) AND (“implant site development” OR “enhancement hard tissue” OR “enhancement soft tissue” OR “implant site regeneration” OR “implant site enhancement”)) OR ((“ bone healing” OR “extraction wound repair” OR “healing extraction socket”) AND (“delayed implant”)).

Once the research was completed, studies were selected in a three-stage process. In the first stage, the reviewers screened the databases, read the article titles, and removed any duplicate studies that did not fit the purpose of the systematic review.

In the second stage, abstracts of all titles approved were downloaded and evaluated; if the abstract met the inclusion criteria, the full text was obtained. When neither the title nor the abstract contained sufficient information for the evaluation, the full text was obtained in any case, to avoid excluding any relevant article. In the last stage, after the collection of all full texts, the inclusion criteria were used to identify the studies that would have been used for the systematic review.

The reviewers performed each phase of this selection process independently, and any disagreement was resolved through discussion. All reviewers agreed on the final selection of the articles.

### 2.2. Inclusion Criteria

- Human studies;
- English language;
- Type of studies: case reports, case series, randomized controlled trials, prospective studies;
- Population (P): subjects needing ridge regenerative procedures prior to implant rehabilitation;
- Intervention (I): implant site development through orthodontic extrusion or regenerative surgery (ridge splitting, guided bone regeneration, distraction osteogenesis, bone and soft tissue grafting);
- Control (C): spontaneous bone healing after tooth loss and delayed implant placement;
- Outcome (O): primary outcome was changes in the amount of bone and soft tissue in the implant site.

### 2.3. Secondary Outcome

- Esthetic outcome;
- Implant survival rate;
- Complications.

### 2.4. Data Extraction

Data regarding the following parameters were extracted:

- Type of study;
- Year;
- Author;
- Patient age;
- Patient gender;
- Health conditions and smoking habit;
- Treated teeth;
- Reason for treatment need;
- Type of treatment (orthodontic, surgical, absent);
- Orthodontic technique: type of appliance, extrusion method, the intensity of the force, anchorage, extrusion ratio (mm/month), duration of the orthodontic treatment (months), frequency of follow-up (weeks), post-orthodontic retention (months), amount of tooth extrusion (mm), amount of bone augmentation (mm), amount of soft tissue augmentation (mm), efficacy of orthodontic extrusion in hard and soft tissue regeneration (%);
- Regenerative surgery: type of treatment (GTR, GBR, bone graft, distraction osteogenesis, ridge-splitting), materials and membranes used, and type of defects;
- Hard tissue changes;
- Soft tissue changes;
- Timing of implant placement;
- Type of implant;
- Treatment outcomes;
- Complications;
- Follow-up data;
- Implant survival rate.

### 2.5. Methodological Quality and Risk of Bias Evaluation

The methodological quality and the risk of bias of randomized and non-randomized studies were evaluated using specific tools designed by the Cochrane Library:

- ROBINS-I tools for non-randomized studies (Prospective studies, Case series);
- Rob 2.0 for Randomized Controlled Trials (RCT).

Quality evaluation of case reports was performed using CARE guidelines instead. Case report guidelines consist of a list of 13 key clinical components that should be present in a well-performed case report.

The reviewers decided to use this list to evaluate the studies. Each key component was given a score; to estimate the quality of each case report, the reviewers read the article evaluating the respect of these key components. For every component, the reviewers gave a score. Finally, all the points were added to obtain the final score of the study.

**Figure 1:**
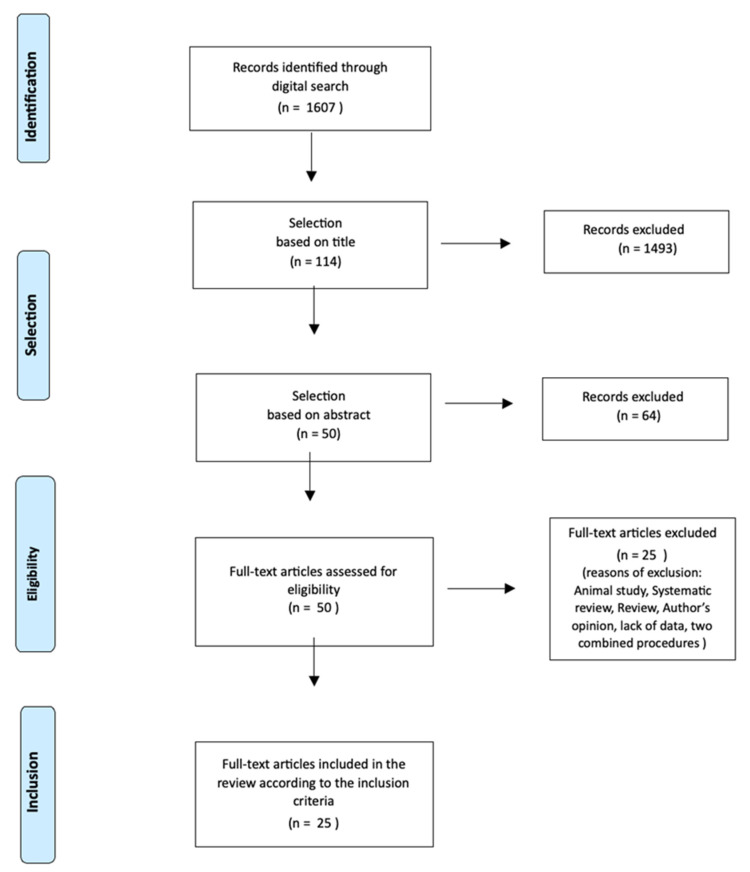
Study flow chart.

Four arbitrary numeric ranges were decided to give a qualitative description of the quality of case reports (excellent, good, sufficient or poor) (Figure 1).

## 3. Results

### 3.1. Characteristics of the Included Studies

In total, 25 studies were included in the review: 11 studies about orthodontic extrusion, 9 studies about regenerative procedures and 5 studies about spontaneous healing of the extraction socket and delayed implant placement.

Eleven studies (9 case reports and 2 case series) reported on orthodontic extrusion and nine studies (5 case reports, 3 case series and 1 RCT) reported on regenerative procedures for implant site development.

Five studies (4 RCT and 1 prospective study) reported on spontaneous healing of the extraction socket and delayed implant placement (Table 1).

The studies were conducted in a private office or in a university setting.

In total, 27 patients were treated by orthodontic extrusion, 103 patients received regenerative procedures and 204 patients were included in the studies comparing spontaneous tissue healing after extraction with ridge preservation procedures.

### 3.2. Characteristics of the Participants

The age of patients treated with orthodontic extrusion ranged from 23 to 62 years. The two case series [5,11] reporting on orthodontic implant site development did not include any data on patients’ age.

The age of patients undergoing regenerative procedures ranged from 18 to 68 years. One case series [24] did not report any information about the patients’ age.

The age of patients included in the randomized studies comparing ridge preservation procedures with spontaneous healing after tooth loss ranged from 20 to 76 years.

In total, 16 patients (59.3%) treated with orthodontic extrusion were women, while only 6 (22.2%) were men; one study [5] did not report on patient sex.

In total, 47 patients (45.6%) treated with regenerative procedures were men, while 46 (44.6%) were women; one study [24] did not report on patient sex.

In total, 77 patients (37.2%) included in the studies comparing spontaneous tissue healing after extraction with ridge preservation procedures were men, while 127 (62.3%) were women (Table 2, Table 3 and Table 4).

Regarding the health of patients treated with orthodontic extrusion, it was reported that 17 patients were healthy, whereas there was no information about the remaining 10 patients’ health conditions.

In total, 91 of the patients treated with regenerative procedures were healthy, 1 patient had high blood pressure and 1 patient was asthmatic; there was no information about the health of the remaining 10 patients.

All the 204 patients included in the randomized studies comparing ridge preservation procedures with spontaneous healing after tooth loss were healthy.

The information provided about the smoking habit of most of the patients treated with orthodontic extrusion was insufficient. Regarding the patients treated with regenerative procedures, both smokers and nonsmokers were included in the studies, but not all studies provided enough information about that aspect.

Patients included in the randomized studies comparing ridge preservation procedures with spontaneous healing after tooth loss were smokers and nonsmokers; only one study [22] did not provide any information about patients smoking habit.

All teeth extruded orthodontically were identified concerning the location: 40 maxillary incisors, 3 maxillary canines, 5 maxillary premolars, 2 maxillary first molars, 4 mandibular incisors, and 1 mandibular first molar.

In the regenerative procedures, most treated teeth were maxillary incisors; two studies [18,19] reported regenerative procedures in the mandibular right posterior region and one study [24] in different mandibular areas, according to the patients’ need.

In the studies comparing spontaneous tissue healing after extraction with ridge preservation procedures, tooth extractions were performed both in maxillary and mandibular arches and in different areas, according to each patient’s need.

The most common reason for the extraction of teeth treated with orthodontic extrusion was severe periodontitis, followed by non-restorable tooth. The reasons for extraction in those patients treated with regenerative procedures were different: periodontitis, non-restorable teeth, fractures, endodontic complications, trauma; three studies [15,24,25] did not provide details regarding the reasons of tooth loss.

In the randomized studies comparing spontaneous tissue healing after extraction with ridge preservation procedures, no details were given about the reason for the extractions.

In the orthodontic extrusion group of studies, information about the type of bone defect affecting the tooth to be extruded was provided in just one case series [11], whereas the other studies only referred to the presence of horizontal and/or vertical defects due to periodontitis.

Most studies about regenerative procedures gave generic information about bone defects, just referring to the presence of severe alveolar bone defects; one study [16] provided the type of defect (Cadwood and Howell class V defect).

In the studies comparing spontaneous tissue healing after extraction with ridge preservation procedures, no details were given about bone defects.

### 3.3. Types of Treatment

#### 3.3.1. Orthodontic Implant Site Development

Regarding the orthodontic implant site development, the most used appliance to perform orthodontic extrusion was a multi-bracket system; in only one study [9], a removable appliance was used. The multi-bracket approach was full bimaxillary in three studies [14,29,30] and partial in seven studies [5,7,8,10,11,12,13].

In one study [8], a lingual appliance was used; in another study [13], ceramic brackets were used (Table 2).

Different ways were employed to apply the extrusive force: three studies [8,13,29] used NiTi archwires and a more apical bonding of brackets on the teeth to be extruded; one study [9] used elastic bands from orthodontic hooks bonded to the cervical region of teeth to be extruded and a removable appliance; one study [14] first used NiTi archwires and a more apical bonding of brackets, then an SS archwire with extrusion steps and intermaxillary elastics; three studies [10,11,12] used SS archwires and extrusion steps; one study [7] used a Co-Cr archwire with extrusion steps, L-loops and inset component for palatal movement; and one study [30] used an SS archwire with L-shape loops.

A low extrusive force (<100 g) was applied in all studies, but five studies [5,8,12,29,30] did not report data about the intensity of the force used to perform the extrusion. Regarding the extrusion ratio, the extrusion was performed slowly (mean of 1 mm/month) in six studies [7,8,9,10,11,30], whereas the other five studies did not report any data.

Just two studies [7,11] reported the amount of tooth extrusion performed.

Most of the studies used an SS archwire as an anchorage during the extrusion; one study [9] used a removable appliance and one study [8] used a composite resin veneer pontic, while three studies did not report information about the anchorage used.

The frequency of follow-up varied from 2 to 4 weeks in all studies.

In all studies but one [14], a stabilization period followed the orthodontic extrusion. The retention period lasted from 1.5 to 6 months, depending on each study.

Tooth extraction was followed by immediate post-extractive implant placement in all studies, except two [9,12], where a delayed implant placement was performed 4–6 weeks after extraction.

All studies but one [5] gave details about the type of implants used.

Eight studies performed a follow-up from a minimum of 6 months [10] to a maximum of 6 years [29]; three studies [5,8,12] did not report information about the duration of follow-up. No complications emerged in all studies except two: in one study [11], one implant failed and gingival recessions (0.1–0.2 mm) were observed, whereas in another study [9], external root resorption and an interdental papillae deficiency were observed.

**Table 2:** Studies on orthodontic implant site development. NR: Not reported.

Author	Patient Age	Sex	Treated Teeth	Reason of Extraction	Type of Appliance	Extrusion Method	Intensity of the Force	Anchorage	Extrusion Ratio	Follow-Up Weeks	Retention (month)	Amount of Tooth Extrusion (mm)	Amount of Bone Augmentation (mm)	Amount of Soft Tissue Augmentation (mm)	Efficacy % Bone	Efficacy % Soft Tissue
Joo, Son, Lee [10]	46	F	1.3	Periodontitis	Multi-bkt appliance.	SS archwire with extrusion steps	10–15 g	Steel archwire	1 mm/month	4	2	NR	NR	2 mm of overcorrection of the gingival margin	NR	NR
Watanabe et al. [7]	41	M	1.1	Periodontitis	Partial multi-bkt from 15 to 25. Micro-arch appliances Formula-R, Roth Type; Tomy Intl Inc.	Co-Cr Archwire with extrusion step, L-loop and inset component for palatal movement. Then TMA archwire	15–80 g	NR	1.4 mm/month	4	2.5	7	NR	6–7 mm	NR	NR
Amato, Mirabella et al. [11]	NR	3 M 10 F	n° 32 teeth, mainly anterior teeth	Periodontitis or non restorable teeth	Multi-bkt appliance.	SS archwire with extrusion steps and finally TMA	15–50 g	Steel archwire	1 mm/month	4	2	3–10 mm	0.6–8.0 mm	1.7–7.7 mm	70%	65%
S. Hyun Kim et al. [9]	30	F	1.1–1.2	Periodontitis	Removable appliance and orthodontic hooks bonded to the cervical region of 1.1 and 1.2	Elastic bands from the bonded hooks and the removable appliance	70–100 g	Removable appliance	1 mm/week followed by 3 weeks of stabilization	4	1.5	NR	NR	NR	NR	NR
Paolone et al. [8]	57	M	2.1	Trauma	Partial lingual multi-bkt from 14 to 24 (Ormco 7th gen).	Cu-NiTi archwire then TMA. More apical bonding.	NR	composite resin 12-11-X-22 veneer pontic	0.5 mm/month	2	6	NR	NR	NR	NR	NR
Holst et al. [13]	23	F	2.1	Non-restorable	Partial ceramic multi-bkt from 14 to 24	NiTi archwire. More apical bonding.	30–50 g	NR	NR	4	3	NR	NR	NR	NR	NR
Erkut et al. [14]	62	F	1.4–1.5 1.6	Periodontitis	Full bimaxillary multi-bkt appliance	NiTi archwire. More apical bonding. Then SS archwire with extrusion steps and inter-maxillary elastics	<100 g	Steel archwire	NR	2	None	NR	NR	NR	NR	NR
Mantzikos, Shamus [12]	34	M	1.1–2.1	Periodontitis	Partial multi-bkt appliance from 13 to 23	SS archwire with extrusion steps, then NiTi archwire	NR	NR	NR	4	4–6	NR	7–8 mm	5 mm	NR	NR
de Barros et al. [29]	56	F	1.1–2.1	Periodontitis	Full bimaxillary multi-bkt appliance	NiTi archwire. More apical bonding	NR	Steel archwire	NR	2 weeks	1	NR	NR	NR	NR	NR
Chou et al. [30]	40	F	4.6	Periodontitis	Full bimaxillary multi-bkt appliance	SS archwire with L-shape loop	NR	Steel archwire	0.5 mm/month	1 month	6 months	NR	8 mm increase in the level bone on the mesial side; 6 mm increase on the distal side	NR	NR	NR
Mantzikos and Shamus [6]	NR	NR	1.1–2.1	Periodontitis	Partial multi-bkt appliance from 13 to 23	SS archwire. More apical bonding	NR	Steel archwire	NR	2 weeks	3 months	NR	Mean 8 mm	NR	NR	NR

Two studies [7,11] only reported the implant survival rate (100% and 96% respectively).

All studies referred to good treatment outcomes, with satisfying esthetic and biological results and a successful implant osteointegration, but only a few [5,7,11,12,30] gave quantitative data about the amount of bone and/or soft tissue augmentation and only one study [11] indicated the percentage of efficacy of the technique in the regeneration of bone and soft tissue in the future implant site (70% and 65%, respectively).

#### 3.3.2. Regenerative Procedures for Implant Site Development

For implant site development, different regenerative procedures were used: four studies [15,17,19,20] performed a guided bone regeneration (GBR), in particular, two studies [15,19] used a one-stage GBR (implant placement and simultaneous bone regeneration), one study [20] used a two-stage GBR (bone regeneration and delayed implant placement in the regenerated bone) and another study [17] divided the patients in two groups and used both types of GBR, one-stage GBR in one group and two-stage GBR in the other one; three studies [16,18,25] performed a bone graft procedure, in particular, one study [18] used an allogenic block graft, whereas the other two studies [16,25] used autogenous bone grafts from an intra-oral site; finally two studies [24,27] performed an alveolar distraction osteogenesis (Table 3).

All studies gave information about the materials used for the regenerative procedures: for GBR procedures, one study [15] used a deproteinized bovine bone mineral (Bio-Oss), one study [17] used a freeze-dried mineralized bone allograft, and the other two studies [19,20] used demineralized freeze-dried bone particles (Dembone); for bone grafting, one study [18] used a Puros block allograft rehydrated with platelet-rich plasma and additional Puros particulate mineralized bone graft material, and the other two studies used autogenous bone grafts from symphysis [25] and from retromolar area [16]; and the studies [24,27] performing alveolar distraction osteogenesis used the same type of distraction device (Modus distractor, Medartis).

All studies performing GBR or bone graft procedures used barrier membranes: three studies [15,16,18] used resorbable membranes and the other four studies [17,19,20,25] used non-resorbable membranes.

Regarding the timing of implant placement, two studies [15,19] performed a regenerative procedure with simultaneous implant placement, two studies [17,25] performed a regenerative procedure with simultaneous or delayed implant placement, depending on each patient’s clinical condition, and five studies [16,18,20,24,27] performed a regenerative procedure with delayed implant placement in the regenerated bone.

All studies except two [16,20] gave details about the type of implants used.

The follow-up period ranged from a minimum of 6 months [15] to a maximum of 3 years [16,24,25], depending on each study.

All studies reported good outcomes, with an enhancement of hard tissue, but none of them gave quantitative data about the amount of regenerated bone. Regarding soft tissue changes, no study reported data, except one [24], using an alveolar distraction osteogenesis procedure which referred to a simultaneous lengthening of soft tissues surrounding the distracted bone segment.

**Table 3:** Studies on regenerative surgery procedures for implant site development.

Author	Patient Age	Sex	Treated Teeth	Type of Defect	Type of Treatment	Materials	Type of Membranes	Follow-Up	Hard Tissue Changes	Soft Tissue Changes	Complications	Implant Survival Rate
Xi Jiang et al. [15]	From 20 to 52 years	15 M 13 F	Maxillary incisors	NR	Implant placement using submerged or transmucosal surgical technique and simultaneous GBR	Deproteinized bovine bone mineral (Bio-Oss)	resorbable collagen membrane (Bio-Gide)	1 week 1 month 6 months Radiographic evaluations	GBR with resorbable collagen membrane and particulate bovine bone undergoes some horizontal volume reduction during the healing stage. Greater reduction happens in the coronal region. The use of different implant healing strategies (transmucosal or submerged) doesn’t make significant differences.	NR	None	100%
J.P. Sullivan [16]	32	F	1.1	Cawood and Howell class V defect.	Autogenous bone graft from an intraoral site	Bone block from the right retromolar area and bovine derived bone particulate (Bio Oss) to fill the gaps	Resorbable porcine derived membrane (Bio-Gide)	3 years	After 3 months the bone block was well integrated	NR	None	NR
Fagan et al. [17]	From 27 to 51 years	18 M 19 F	Anterior maxilla	Absence or loss >50% of the buccal plate and gingival recession or thin gingival biotypes	Bone graft, use of platelet-derived growth factors and pediculated connective tissue graft to simultaneously augment hard and soft tissue. Immediate or delayed implant placement.	Freeze-dried mineralized bone allograft, recombinant human platelet derived growth factor mixture	titanium-reinforced membrane	1 year	Enhancement of hard tissue	Enhancement of soft tissue	None	97.3%
Petrungaro et al. [18]	55 years and 26 years	2 F	Mandibular right posterior region (Case report 2) and maxillary anterior region (Case report 3)	Advanced bone loss in the mandibular right posterior region (Case report 2) and in the maxillary anterior region (Case report 3)	Allogenic block graft and delayed implants	Puros block allograft rehydrated with platelet-rich plasma and additional particulate mineralized bone graft material (Puros)	Tutoplast Processed Pericardium; Tutogen Medical	1 year	Enhancement of hard tissue	NR	None	NR
Artzi et al. [19]	From 42 to 54 years	1 M 3 F	1.3–1.1 4.4 and all the upper left quadrant	Different amount of bone loss	Immediate post-extractive implant placement and simultaneous GBR	Demineralized freeze-dried bone particles (Dembone)	e-PTFE membrane	2 years	Enhancement of hard tissue with histological evaluations.	NR	None	NR
Artzi et al. [20]	From 32 to 40 years	3 F	1.6 1.5–1.4 1.2–1.1 2.1–2.2	Different amount of bone loss	GBR and delayed implant placement	Demineralized freeze-dried bone particles (Dembone)	e-PTFE membrane	2 years	Enhancement of hard tissue	NR	Early exposure of the e-PTFE membrane in one case, but with no consequences	NR
Sezer et al. [24]	NR	NR	Distraction was performed in different mandibular areas, according to each patient need.	Severe alveolar bone atrophy in mandible	Alveolar Distraction Osteogenesis	Distraction device (Modus ARS 1.5 V2 Distractor; Medartis)	None	3 years	Increase in alveolar bone height with new bone formation beneath the distracted bone. Mean bone gain after distraction: 7mm	Simultaneous lengthening of the surrounding soft tissues.	In 1 patient a progressive lingual inclination of the distracted segment occurred during distraction. In 1 patient, a transient paresthesia of the area innervated by the inferior alveolar nerve was observed. Infection was observed in 1 case in the postoperative period.	100%
McCarthy et al. [25]	From 18 to 68 years	12 M 5 F	Anterior maxilla	Different amount of bone loss in the anterior maxilla.	Onlay Bone Grafts from the mandibular symphysis to anterior maxilla.	Corticocancellous block grafts and particulate cancellous grafts from the mandibular symphysis.	Nonresorbable Gore-Tex membrane	3 years	Enhancement of hard tissue and improvement of the esthetic outcome.	NR	4 patients reported paresthesia at the donor site immediately following the graft surgery.	97.1%
Gozneli et al. [27]	40	M	1.2–1.1 2.1–2.2	Severe alveolar bone loss and gingival recessions in anterior maxilla.	Alveolar Distraction Osteogenesis	Distractor device (Modus Distractor; Medartis)	None	1.5 years	An adequate vertical bone volume was obtained.	An adequate soft tissue volume around implants was obtained.	None	NR

Only three studies [17,24,25] indicated the implant survival rate (100%, 97.3% and 97.1%, respectively).

Only three studies [20,24,25] reported complications: one study [24] using alveolar distraction osteogenesis referred to a lingual inclination of the distracted bone segment occurred during distraction in one patient and a post-operative infection in another patient; one study [20] using a GBR technique reported an early exposure of the e-PTFE membrane; finally, one study [25] using an onlay bone graft from the mandibular symphysis reported four cases of paresthesia at the donor site immediately following the graft surgery.

#### 3.3.3. Spontaneous Healing of the Extraction Socket and Delayed Implant Placement

Regarding the delayed implant placement in an untreated edentulous ridge, five studies were included in the systematic review: one prospective study [21] analyzed the spontaneous healing of the extraction socket and a delayed implant placement; the other four randomized controlled studies [22,23,26,28] compared extraction alone with ridge preservation procedures before delayed implant placement (Table 4).

**Table 4:** Studies on spontaneous healing of the extraction socket and delayed implant placement.

Author	Patient Age	Sex	Type of Treatment	Treated Teeth	Timing of Implant Placement	Follow-Up	Hard Tissue Changes	Soft Tissue Changes	Complications	Implant Survival Rate
Crespi et al. [21]	From 43 to 70 years	14 M 26 F	Atraumatic tooth extraction. Spontaneous healing of the extraction socket. Granulation tissue left in situ. No graft procedure.	Maxillary molars	Delayed implant placement 3 months after extraction	3 months, 3 years	Bucco-lingual width showed a statistically significant decrease at implant placement. Moreover, a statistically significant increase was measured 3 years after implant insertion. Not statistically significant differences were found between baseline values (before extraction) and at 3 years from implant placement (11.44 ± 1.80 mm and 11.59 ± 1.61 mm, respectively). Vertical dimension showed statistically significant increase between baseline values (before extraction) and at 3 years from implant placement (8.05 ± 2.12 mm and 12.48 ± 2.04 mm, respectively)	NR	NR	100%
Iasella et al. [22]	From 28 to 76 years	10 M 14 F	Ridge Preservation procedure for implant site development with freeze-dried bone allograft and a collagen membrane compared to extraction alone	Teeth extracted consisted of: 11 maxillary premolar, 6 maxillary incisors, 1 maxillary canine and 6 mandibular premolars.	Delayed implant placement 4–6 months after extraction	6 months	Ridge preservation procedures improved ridge height and width dimensions when compared to extraction alone. The width of the RP group decreased from 9.2 ± 1.2 mm to 8.0 ± 1.4 mm, while in the EXT group decreased from 9.1 ± 1.0 mm to 6.4 ± 2.2 mm, a difference of 1.6 mm. Most of resorption occurred from the buccal. The vertical change for the RP group was a gain of 1.3 ± 2.0 mm versus a loss 0.9 ± 1.6 mm for the EXT group. Histologic analysis revealed more bone in the RP group.	On the buccal aspect the RP group lost soft tissue thickness (−0.1 ± 0.5 mm), while the EXT group gained thickness (0.4 ± 0.6 mm)	Some sites showed little dehiscences at the time of implant placement	NR
Barone et al. [23]	From 26 to 69 years	16 M 24 F	Xenograft versus Extraction Alone for Ridge Preservation after tooth extraction.	Tooth extractions were performed both in maxillary than in mandibular arch, according to each patient need.	Delayed implant placement, 7 months after extraction.	7 months	A significantly greater horizontal resorption was observed at EXT sites (4.3 ± 0.8 mm) compared to RP sites (2.5 ± 1.2 mm). The ridge height reduction at the buccal side was 3.6 ± 1.5 mm for the extraction-alone group, whereas it was 0.7 ± 1.4 mm for the ridge-preservation group. Moreover, the vertical change at the lingual sites was 0.4 mm in the ridge-preservation group and 3 mm in the extraction-alone group. The histologic analysis showed a significantly higher percentage of trabecular bone and total mineralized tissue in ridge-preservation sites compared to extraction-alone sites	NR	None	NR
Barone et al. [28]	From 20 to 63 years	20 M 38 F	Spontaneous healing vs. ridge preservation with secondary soft tissue healing	Tooth extractions were performed both in maxillary than in mandibular arch, according to each patient need.	Delayed implant placement, 4 months after tooth extraction	8 months	In the grafted group changes in horizontal dimension showed an average resorption of 1.6 ± 0.55 mm. Vertical bone resorption was 0.3 ± 0.76 mm, 1.1 ± 0.96 mm, 0.3 ± 0.85 mm, 0.9 ± 0.98 mm at the mesial, buccal, distal and lingual sites respectively. In the non-grafted group, horizontal bone resorption was significantly higher (3.6 ± 0.72 mm). Vertical measurements indicated an average resorption of 1 ± 0.7 mm, 2.1 ± 0.6 mm, 1 ± 0.8 mm and 2 ± 0.73 mm respectively, at the mesial, buccal, distal and lingual sites.	The width of keratinized gingiva was better preserved in the grafted group. A greater shift of the gingival tissue towards the occlusal direction (mean 1.1 ± 0.9 mm) was observed in the grafted sites when compared to non-grafted sites (mean 0.7 ± 0.8 mm).	None	NR
Marconcini, Covani et al. [26]	mean of 53 years	17 M 25 F	Ridge preservation with cortical (CORT) or collagenated (COLL) corticocancellous porcine bone compared to extraction alone (EXT).	According to each patient need: 1 tooth among first molars, first and second premolars or canines of both arches.	Delayed implant placement, 3 months after extraction.	4 years	There were no differences regarding marginal bone change between the collagenated (COLL) and the cortical (CORT) corticocancellous porcine bone groups. Both grafts seemed to preserve the peri-implant marginal bone better than the natural healing. The total amount of marginal bone loss from T0 to T4-year was 1.14 ± 0.23 mm in the CORT group, 1.13 ± 0.29 mm in the COLL group and 1.92 ± 0.07 mm in the EXT group.	The Pink Esthetic Score (PES) resulted significantly better (9.42 ± 0.75) for the CORT group than for the COLL group (8.53 ± 1.18) and EXT group (6.07 ± 1.89) at 4-year evaluation.	None	100%

In the prospective study [21], no ridge preservation procedure was performed after tooth extraction, but the extraction was as atraumatic as possible, and the granulation tissue was left in situ to help the healing process. Implants were placed three months after tooth extraction.

In the RCT studies ridge preservation procedures were performed after tooth extraction to limit the resorption of hard and soft tissues: one study [22] used a freeze-dried bone allograft and a collagen membrane; one study [23] used a xenograft; one study [26] used a cortical or collagenated corticocancellous porcine bone graft; finally, one study [28] used a corticocancellous porcine bone graft and a collagen membrane, which remained exposed in the oral environment for an intentional secondary soft tissue healing. A delayed implant placement followed all these ridge preservation procedures: in one study [26], implants were inserted 3 months after tooth extraction; in two studies [22,28] implants were placed 4 months after extraction; and in another study [23], implants were inserted 7 months after tooth extraction.

Two studies [21,26] only reported data about the type of implants used.

The follow-up period was different: in three studies [22,23,28], it was 6, 7, and 8 months long, respectively, 3 years in the prospective study [21], and 4 years in another randomized study [26].

Regarding complications, only one study [22] reported the presence of little dehiscences at the time of implant placement in the group of patients treated with ridge preservation procedures. The other studies reported no complications.

All studies reported data about hard tissue changes. In the prospective study [21], no statistically significant differences were found in the ridge width before the extraction and 3 years after implant placement (11.44 ± 1.80 mm and 11.59 ± 1.61 mm, respectively), whereas the vertical ridge dimension showed a statistically significant increase (from 8.05 ± 2.12 mm to 12.48 ± 2.04 mm). The RCT studies showed that all ridge preservation procedures limited the horizontal and vertical resorption of hard tissue after tooth extraction, compared to extraction alone. In one study [22], the ridge width decreased from 9.2 ± 1.2 mm to 8.0 ± 1.4 mm in the group treated with ridge preservation procedures (RP), while it decreased from 9.1 ± 1.0 mm to 6.4 ± 2.2 mm in the extraction-alone (EXT) group, a difference of 1.6 mm. Most of resorption occurred from the buccal. The vertical change for the RP group was a gain of 1.3 ± 2.0 mm versus a loss of 0.9 ± 1.6 mm for the EXT group. Histologic analysis revealed more bone in the RP group.

In another study [23], a significantly greater horizontal resorption was observed at EXT sites (4.3 ± 0.8 mm) compared to RP sites (2.5 ± 1.2 mm). The ridge height reduction at the buccal side was 3.6 ± 1.5 mm for the EXT group, whereas it was 0.7 ± 1.4 mm for the RP group. Moreover, the vertical change at the lingual sites was 0.4 mm in the RP group and 3 mm in the EXT group. The histologic analysis showed a significantly higher percentage of trabecular bone and total mineralized tissue in ridge-preservation sites compared to extraction-alone sites.

One study [28] reported that in the grafted group changes in horizontal dimension showed an average resorption of 1.6 ± 0.55 mm. Vertical bone resorption was 0.3 ± 0.76 mm, 1.1 ± 0.96 mm, 0.3 ± 0.85 mm, 0.9 ± 0.98 mm at the mesial, buccal, distal and lingual sites, respectively. In the non-grafted group, horizontal bone resorption was significantly higher (3.6 ± 0.72 mm). Vertical measurements indicated an average resorption of 1 ± 0.7 mm, 2.1 ± 0.6 mm, 1 ± 0.8 mm and 2 ± 0.73 mm respectively, at the mesial, buccal, distal and lingual sites.

Finally, another study [26] reported that there were no differences regarding marginal bone change between the collagenated (COLL) and the cortical (CORT) corticocancellous porcine bone groups. Both grafts seemed to preserve the peri-implant marginal bone better than the natural healing. The total amount of marginal bone loss from T0 to T4-year was 1.14 ± 0.23 mm in the CORT group, 1.13 ± 0.29 mm in the COLL group and 1.92 ± 0.07 mm in the EXT group.

Regarding soft tissue changes, two studies [21,23] did not report data, and one study [22] reported a gain in soft tissue thickness in the group of patients treated with extraction alone and a loss of thickness in the group of patients treated with ridge preservation procedures; the other two studies [26,28] reported a better-preserved width of keratinized gingiva in the groups of patients subjected to ridge preservation procedures.

Only two studies [21,26] reported the implant survival rate (100% both).

### 3.4. Methodological Quality and Risk of Bias Evaluation

Regarding the methodological quality of studies included in the review, different tools were used to perform the evaluation. For randomized studies [15,22,23,26,28] (RCT), Rob 2.0 tool was used: all studies had good quality and a low risk of bias (Table 5).

For non-randomized studies [11,17,21,24,25] (case series and prospective study) ROBINS-I tool was used: two studies [11,24] only had a low risk of bias, whereas the other three studies [17,21,25] had a moderate risk of bias (Table 6).

For the evaluation of case reports, the CARE guidelines were used: two studies [10,27] only had a good quality, three studies [9,29,30] had sufficient quality, and the remaining studies [7,8,12,13,14,16,18,19,20] had a poor methodological quality (Table 7).

## 4. Discussion

Regarding orthodontic extrusion for implant site development, most studies [7,8,9,10,12,13,14,29,30] were case reports, except two, which were case series [5,11]. The studies reporting on regenerative procedures for implant site development included five case reports [16,18,19,20,27], three case series [17,24,25] and one RCT [15]. The majority of studies comparing ridge preservation procedures with spontaneous healing after tooth loss were RCT [22,23,26,28,31], except one, which was a prospective study [21].

Patients undergoing orthodontic extrusion ranged from 23 to 62 years old; patients treated with regenerative surgery ranged from 18 to 68 years old; and, finally, patients included in the studies comparing spontaneous tissue healing versus extraction alone ranged from 20 to 76 years old.

The mean age of patients treated with orthodontic extrusion or regenerative procedures was 40 years, and it was a little lower than the one of patients included in the studies about spontaneous tissue healing after tooth loss and delayed implant placement (52 years); it is not known if this was due to the fact that older patients prefer not to submit to surgery or orthodontic procedures prior to implant placement, or to the clinician’s preference. In all studies, most of the patients were women.

The presence of a systematic disease before Implant placement has been discussed in the literature. A retrospective study [31] that investigated the correlation between systemic diseases and implant treatment found a failure rate of 3.6% and concluded that osteoporosis and Crohn’s disease were significantly associated with early implant failure. Furthermore, the same authors [32] concluded that these factors were not associated with late implant loss. The literature suggests that the level of evidence regarding the association between systemic diseases and implant loss is low [33], but the control of any systemic disease before an implant therapy is considered important [34], and any systemic disease in the patients included in the study should be reported. Regarding health conditions, most patients of the studies included in the systematic review were in good health, but not all studies provided enough information on this subject.

Two retrospective studies [35,36] identified a correlation between smoking and implant complications. For what concerns the smoking habit, both smokers and nonsmokers were included in the studies about regenerative procedures and in the studies comparing ridge preservation procedures with spontaneous healing after tooth loss, as soon as the smoking addiction was moderate (fewer than 10 cigarettes per day). The information provided about patient smoking habits in the group of patients treated with orthodontic extrusion was insufficient.

The biological and functional result of implant treatment is very important regardless of the implant’s position; however, the esthetic outcome is fundamental in the anterior maxilla, especially in patients with a high lip line. The quality and quantity of alveolar bone and soft tissue in the future implant site is essential for proper tridimensional implant placement to achieve esthetic and functional outcomes; therefore, several techniques have been employed to develop the implant site. Both mandibular and maxillary anterior and posterior teeth were treated with orthodontic extrusion or regenerative procedures to enhance the implant site, but the majority were anterior maxillary teeth.

Periodontitis was the primary reason for tooth extraction in the studies reporting on orthodontic extrusion, whereas in the studies reporting on regenerative procedures, many causes can be identified, such as periodontitis, non-restorable tooth, traumas, fractures and endodontic complications. The randomized studies comparing spontaneous tissue healing after extraction with ridge preservation procedures provided no details about the reason for the extractions.

Most of studies did not report any data about the type of bone defects affecting the teeth to be treated, except two: one case series [11] about orthodontic extrusion, which reported a classification of the different types of osseous defects based on the amount of the residual periodontal attachment, and another study [16] about regenerative surgery, which reported the type of defect (Cadwood and Howell class V defect). In the studies comparing spontaneous tissue healing after extraction with ridge preservation procedures, no details were given about bone defects.

Although it has been suggested an association between patient’s gingival biotype and the tendency toward gingival recessions after surgical procedures [37,38,39], only three studies [11,17,22] included information on this subject.

The articles reviewed provided detailed information regarding the treatment procedures and materials used. Different methods were employed to apply the extrusive force: NiTi archwire and a more apical bonding of brackets on the teeth to be extruded, SS archwire with extrusion steps, SS archwire with L-shape loops or elastic bands from orthodontic hooks bonded to the cervical region of teeth to be extruded and a removable appliance. The most used appliance to perform orthodontic extrusion was a multi-bracket system; in only one study [9], a removable appliance was used. The multi-bracket approach was full bimaxillary in three studies [14,29,30] and partial in seven studies [5,7,8,10,11,12,13]. In one study [8], a lingual appliance was used; in another study [13], ceramic brackets were used.

No clinical study has proved the ”uper’ority of extrusive biomechanics versus another, but it is mandatory to use low and controlled extrusive forces. A low extrusive force (<100 g) was applied in all studies, but five studies [5,8,12,29,30] did not report data about the intensity of the force used to perform the extrusion. It is important to understand that when tension is applied to the periodontal ligament, periodontal fibers bundles are elongated, and osteoblasts are induced to deposit new bone in the areas where periodontal attachment exists. The same effects on alveolar bone height can be seen when orthodontic treatment is carried out with controlled forces which do not tear apart the periodontal fibers. In other words, a tooth moved into the dental arch by controlled extrusive orthodontic forces will bring alveolar bone, and soft tissues will follow, too [5,6]. Therefore, using low and controlled extrusive forces is fundamental to enhance hard and soft tissues through orthodontic extrusion.

Regarding the extrusion ratio, the extrusion was performed slowly (mean of 1 mm/month) in six studies [7,8,9,10,11,30] whereas the other five studies did not report any data.

Just two studies [7,11] reported the amount of tooth extrusion performed.

Another important aspect has sufficient anchorage to limit the risk of adverse side effects on the teeth adjacent to those planned to be extruded. Most of the studies used an SS archwire as an anchorage during the extrusion; one study [9] used a removable appliance and one study [8] used a composite resin veneer pontic, while three studies did not report information about the anchorage used.

The orthodontic extrusion should be performed according to the tooth’s long axis to avoid generating any apical compression against the buccal cortical bone, which is likely to cause bone fenestrations.

The frequency of follow-up varied from 2 to 4 weeks; during each control visit, hygiene conditions were checked, the orthodontic extrusion device was activated, and progressive occlusal adjustments were performed in order to maintain the teeth extruded out of the occlusal plane.

In all studies except one [14], a stabilization period followed the orthodontic extrusion to ensure a complete maturation of the newly formed bone; the retention period lasted from 1.5 to 6 months, depending on each study and retention was ensured in most of cases by the multibracket system, which was left in place. Tooth extraction was followed by immediate post-extractive implant placement in all studies, except two [9,12], where a delayed implant placement was performed 4–6 weeks after extraction. Immediate post-extractive implant placement is suggested to keep the hard and soft tissue volume obtained through the orthodontic technique.

All studies showed good treatment outcomes, with satisfying esthetic and biological results and successful implant osteointegration, but two studies [7,11] only reported the implant survival rate (100% and 96%, respectively).

Regarding the amount of bone and/or soft tissue augmentation obtained through the orthodontic extrusion, only a few studies [5,7,11,12,30] gave quantitative data, and only one study [11] indicated the percentage of the efficacy of the technique in the regeneration of bone and soft tissue in the future implant site (70% and 65%, respectively). Most studies reported qualitative results, and for this reason, it is not possible to calculate the real efficacy of the orthodontic technique in the regeneration of hard and soft tissues in a future implant site.

No complications emerged in almost all studies. Nevertheless, we must keep in mind that some bias of non-published data may occur in cases with technical failures, and that makes it difficult to establish an accurate success/failure ratio of the employed technique.

At the moment, based on the available results, it is only possible to say that orthodontic extrusion appears as an interesting non-surgical technique to develop a future implant site using hopeless teeth, with apparently fewer disadvantages and risks than the traditional surgical techniques (GBR, ridge splitting, distraction osteogenesis, and bone grafting). The only constraints to the use of this technique are the presence of hopeless teeth to be extruded with a sufficient quantity of residual periodontal attachment and a sufficient anchorage unit [31,32,33,34,35,36,37].

Some recommendations to optimize the clinical results can be given: low and controlled (<100 g) extrusive forces must be used; an extrusion ratio < 1 mm/month should be performed; it is important to use an anchorage on the teeth adjacent to the ones planned to be extruded; the extrusion should be performed according to the long tooth axis; regular follow-up of patients should be scheduled every month; a post-extrusion retention period is suggested; immediate post-extractive implants are suggested to keep the hard and soft tissues volume obtained through the orthodontic technique.

Further studies are necessary to evaluate the efficacy of the technique in the regeneration of hard and soft tissues, to compare the different extrusive biomechanics and to create an evidence-based clinical protocol. Another aspect to investigate is the resorption rate of the orthodontically regenerated bone to compare the quality and quantity of bone regenerated through orthodontic extrusion with that obtained using regenerative procedures.

For implant site development, different regenerative procedures were used: four studies [15,17,19,20] performed a one-stage or two-stage guided bone regeneration (GBR), three studies [16,18,25] performed a bone graft procedure, and two studies [24,27] performed alveolar distraction osteogenesis.

A wide variety of materials were employed for the regenerative procedures, and all studies gave information about the materials used: for GBR procedures, one study [15] used a deproteinized bovine bone mineral (Bio-Oss), one study [17] used a freeze-dried mineralized bone allograft and the other two studies [19,20] used demineralized freeze-dried bone particles (Dembone); for bone grafting, one study [18] used a Puros block allograft rehydrated with platelet-rich plasma and additional Puros particulate mineralized bone graft material, and the other two studies used autogenous bone grafts from symphysis [25] and from the retromolar area [16]; and the studies [24,27] performing alveolar distraction osteogenesis used the same type of distraction device (Modus distractor, Medartis).

All studies performing GBR or bone graft procedures used barrier membranes: three studies [15,16,18] used resorbable membranes and the other four studies [17,19,20,25] used non-resorbable membranes.

Regarding the timing of implant placement, two studies [15,19] performed a regenerative procedure with a simultaneous implant placement, two studies [17,25] performed a regenerative procedure with a simultaneous or delayed implant placement, depending on each patient clinical condition and five studies [16,18,20,24,27] performed a regenerative procedure with a delayed implant placement in the regenerated bone.

All studies except two [16,20] gave details about the type of implants used.

The follow-up period ranged from a minimum of 6 months [15] to a maximum of 3 years [16,24,25], depending on each study.

All studies reported good outcomes, with an enhancement of hard tissue, but few of them gave quantitative data about the amount of regenerated bone; therefore it is not possible to evaluate the efficacy of the different techniques in bone regeneration. Regarding soft tissue changes, no study reported data, except one [24].

Each regenerative surgical procedure showed some limits; one randomized study [15] reported that GBR with resorbable membranes and particulate bone substitutes undergoes some horizontal volume reduction during the healing stage. Greater reduction occurs at the coronal region and the use of different implant healing strategies (transmucosal or submerged) does not make a significant difference. This volume instability could result in the shrinkage of the grafted area and alteration of the ridge contour. To compensate for the volume reduction during the healing stage of GBR, over augmentation has been recommended by some clinicians [40]. In the coronal region, new bone formation may be predictably expected only within the bony envelope of the defect, but further studies are needed to confirm this result. The regenerated hard tissue, determined by the radiographic observation of a radiopacity, may not be true new bone in a histological sense, because not always the biomaterial is resorbed and replaced by new bone, but it can remain as a “foreign body” within the graft [41,42,43,44,45].

Another difficulty with GBR techniques is associated to the use of membranes: non-resorbable membranes have good mechanical properties but they also have a lower biocompatibility, request a second surgery for their removal and there is always a risk of early exposure during the healing process, which can compromise the result. Resorbable membranes have higher biocompatibility and do not request a second surgery, but they have unfavorable mechanical properties, volume instability due to their soft consistency and low resistance to pressure from the surrounding tissues, which may result in membrane collapse and the compromise of the new bone formation due to the loss of tissue volume during the healing stage of the GBR procedure.

Finally, the GBR technique can only regenerate hard tissue, to ease an implant placement, but the soft tissue volume is often inadequate to provide an esthetic result, and it is difficult to simultaneously graft the ridge-augmentation site with soft tissue because of poor vascular support over a GBR membrane. Therefore, the esthetic result sometimes may be unsatisfactory [46].

Autogenous bone is the ideal biomaterial, thanks to its biocompatibility and osteogenicity. Regarding bone graft procedures, autogenous bone blocks remain the ‘gold standard’; intraoral block grafts harvested from the symphysis or the mandibular ramus can be used to reconstruct horizontal and vertical ridge deficiencies prior to implant placement. If the amount of bone needed exceeds what is available intraorally, extraoral sites, such as the iliac crest and calvarium, can be considered [47,48]. The main disadvantage of autologous bone grafts is the morbidity of the donor site; another complicating factor is the additional surgical time necessary to harvest the bone block. As an alternative to this type of grafting, allogenic, xenographic and alloplastic materials have been developed, but they can only provide osteoconduction or osteoinduction, not osteogenesis, and none of them guarantee the healing and bone replacement that autogenous block grafts can provide [49,50,51,52,53,54,55].

The successful integration of a bone graft depends on numerous factors, which are all associated with a good surgical technique. In the receiving site, the cortical plate should be perforated to induce bleeding, which is fundamental to the healing process. There should be intimate contact between the bone block and the recipient site; precise measurements or a template of the recipient site can help to ensure that the harvested bone block is adequate in size and form. The bone graft must be completely still during the healing process, because any movement causes its failure: for this reason, the blocks should be secured through mini screws to the host site. Once the block is screwed, a tension free flap closure and a primary healing must be obtained. Strict asepsis protocols must be respected [56].

Implant primary stability, whenever possible, should be researched in the native bone of the patient, even basal bone: when the native bone ensures the primary stability and the graft only works as a coverage for the implant, implants can be placed 4 months after the graft procedure; otherwise, when there is no residual native bone, it is recommended to wait 6–8 months from the graft procedure before placing the implants, because new bone must form inside the graft in order to have primary stability for the implant. Bone augmentation could also be performed simultaneously with implant placement, but it is associated with increased failure rates [57].

Ridge splitting technique can increase the width of bone by splitting and expanding the existing residual ridge. Indications of this technique include the ridge deficient in width with an adequate amount of height, with no vertical defects. The maxilla is preferred over the mandible because the expansion of bone is easily achieved without any complications thanks to the cancellous nature of bone. Advantages include no second surgery required to place implants simultaneously inserted during the ridge splitting procedure, reduced overall treatment time and increased implant stability because of new bone formation between cortical plates. Disadvantages include more severe ridge defects in case of procedure failure and primary closure being difficult to achieve [58,59,60].

Distraction osteogenesis is based on the principle of secondary wound healing. Osteotomy removes a bone segment from the basal bone, and the distraction gap is filled with callus that forms a new bone segment. This procedure demonstrates many advantages such as decreased bone resorption, no donor site morbidity and simultaneous lengthening of the surrounding soft tissues. This technique requires a delayed implant placement (at least 4 months from the end of the distraction) to wait for the consolidation of distracted bone segments. Therefore, there are also disadvantages, such as the risk of fracture of the mandible, fracture of the transport segment, difficulties in finishing the osteotomy on the lingual side, incorrect direction of distraction, perforation of the mucosa by the transport segment, suture dehiscence, bone formation defects and pain during the activation of the distraction device.

In conclusion, based on the available results, all these regenerative procedures allow an enhancement of a future implant site, with satisfying esthetic and functional outcomes. No study reported a failure and no complications emerged in almost all studies. Nevertheless, we must keep in mind that some bias of non-published data may occur in cases with technical failures, and that makes it difficult to establish an accurate success/failure ratio of the employed techniques [61,62].

Compared to orthodontic extrusion, the regenerative surgical techniques appear more complex, with a higher risk of complications and with a limited ability to develop soft tissues. Regarding the regeneration of hard tissue, most studies reported qualitative results, and for this reason, it is not possible to calculate the real efficacy of the different techniques in the formation of new bone and it is not possible to compare the results with those obtained through orthodontic extrusion [63,64].

Finally, the authors have evaluated randomized studies comparing ridge preservation procedures with spontaneous healing of the edentulous ridge after tooth loss and a delayed implant placement. Overall, the different ridge preservation procedures reduced post-extraction alveolar ridge dimensional changes, compared to extraction alone, but they were unable to prevent resorption. Another systematic review [65] confirmed these results, but also suggested the use of barrier membranes, flap surgical procedures and full flap closure to achieve better results. The obtained results, however, could not indicate which type of surgical procedure or biomaterial is most suitable for this approach.

Regarding hard tissue volume, even if ridge preservation procedures appear to be better than extraction alone followed by a delayed implant placement, one prospective study [21] showed that, if tooth extraction is atraumatic and the granulation tissue is left in situ, both horizontal and vertical dimension could be maintained, because the granulation tissue contains new small blood vessels and pluripotent stem cells that contribute to tissue healing. Regarding soft tissue volume, the authors found discordant results. One study [22] reported a loss of soft tissue thickness on the buccal aspect in the ridge preservation group of patients. Kirkland et al. previously showed that using a resorbable membrane for ridge augmentation decreased soft tissue thickness; the same phenomenon occurred in this study. Soft tissue thickness loss occurs together with hard tissue loss; the greatest loss is on the buccal side; therefore, it has important relevance in the esthetic zone. This loss of soft tissue thickness is most likely due to the interference with flap vascularity by the membrane and the graft; since the membrane is interposed between the flap and the bone surface, the vascular supply for the flap comes only from the flap base rather than the dual blood supply from the underlying osseous and flap base found in non-grafted sites. On the contrary, two other studies [26,28] reported a greater soft tissue volume in the grafted groups. Further studies are needed to confirm these results, and split-mouth studies would be the most indicative to compare these different approaches.

### Study Limitations

The methodological quality evaluation showed that most of studies regarding orthodontic extrusion are case reports, which correspond to the lowest level of scientific evidence; moreover, the lack of quantitative data about the outcomes of the different approaches limits the strength of the conclusion that can be drawn and does not allow the creation of an evidence-based protocol for the development of the implant site. In this perspective, different methods under different conditions could lead to difficult comparison evaluation.

## 5. Conclusions

The present systematic review demonstrated that there is a substantial lack of data and evidence to determine which of the presented methods is better to develop a future implant site; the main problem is the absence of randomized controlled trials and multicenter studies comparing the two implant site development approaches: orthodontic extrusion versus regenerative surgery. On the contrary, in the literature, there are many RCT studies comparing ridge preservation procedures and extraction alone, but further studies are needed to identify the best materials and procedures to use and to create an evidence-based protocol for clinicians.

Based on the available results, both surgical and non-surgical procedures appear effective in the regeneration of hard tissue in a future implant site, whereas not all the techniques can improve soft tissue volume, too.

Regarding orthodontic extrusion, no clinical study has proved the superiority of extrusive biomechanics versus another yet, but some useful recommendations can be given to clinicians:

- Low and controlled (<100 g) extrusive forces;
- Extrusion ratio < 1 mm/month;
- Extrusion according to the tooth long axis;
- A post-extrusion retention period is suggested to wait for the maturation of the regenerated bone;
- Immediate post-extractive implants are suggested to keep the hard and soft tissues volume obtained through the orthodontic technique; a guided insertion could be beneficial for aesthetic and patient comfort.

## Figures and Tables

**Table 1:** Studies included in the systematic review. NR, not reported.

Author	Year	Type of Study	Type of Treatment	Treatment Outcomes	Timing of Implant Placement	Type of Implant	Follow-Up	Complications	Implant Survival Rate
Joo, Son, Lee [10]	2016	Case report	Orthodontic extrusion	Enhancement of hard and soft tissue and successful implant placement	Immediate post-extractive implant	4.0 × 11.5 mm Nobel Biocare	6 months	None	NR
Watanabe et al. [7]	2013	Case report	Orthodontic extrusion	Successful implant therapy. Satisfying esthetic and biologic results	Immediate post-extractive implant	3.75 × 15 mm implant Nobel Mk III Nobel Biocare	4 years	None	100%
McCarthy et al. [25]	2003	Case series (17 patients)	Onlay Bone Grafts from the mandibular symphysis to anterior maxilla.	Satisfying results	Immediate or delayed implants	Brånemark System Mark II implants (Nobel Biocare)	3 years	4 patients reported paresthesia at the donor site immediately following the graft surgery.	97.1%
Amato, Mirabella et al. [11]	2012	Case series (13 patients)	Orthodontic extrusion	Successful implant therapy. Satisfying esthetic and biologic results	Immediate post-extractive implant	3.25, 4.0, 5.0 × 10, 11, 13, 15 mm. Biomet	1.5–5 years	1 implant failed ,Recessions 0.2–5.7 mm	96%
S. Hyun Kim et al. [9]	2011	Case report	Orthodontic extrusion	Successful implant placement and improvement of esthetics	6 weeks post extraction	3.75 × 11 screw-type machined- submerged implants Titamax Ti Cortical; Neodent,	5 years	Interdental papillae deficiency around 1.1 and 1.2 Some external root resorption in the apical third of 1.2	NR
Marconcini, Covani et al. [26]	2018	RCT (42 patients)	Ridge preservation procedure with cortical (CORT) or collagenated (COLL) corticocancellous porcine bone compared to extraction alone (EXT)	Ridge preservation was more effective than natural healing in preserving marginal bone and in achieving esthetic outcomes around implants. The CORT showed better clinical outcomes than COLL.	3 months after extraction	Delayed implants (BT Evo; Biotec)	4 years	None	100%
Paolone et al. [8]	2008	Case report	Orthodontic extrusion	Successful implant therapy. Satisfying esthetic and biologic results	Immediate post-extractive implant	4.5 × 15 mm root-form implant FRIALIT-2 Dentsply FRIADENT	NR	None	NR
Holst et al. [13]	2007	Case report	Orthodontic extrusion	Successful implant rehabilitation and good esthetic outcomes	Immediate post-extractive implant	4 × 13 mm implant Nobel Biocare	2 years	None	NR
Erkut et al. [14]	2007	Case report	Orthodontic extrusion	Successful implant therapy. Satisfying esthetic and biologic results	Immediate post-extractive implant	4.1 × 12 mm implants	7 months	None	NR
Mantzikos and Shamus [5]	1999	Case series (5 patients)	Orthodontic extrusion	Enhancement of soft and hard tissue and successful implant placement	Immediate post-extractive implant	NR	NR	None	NR
Mantzikos, Shamus [12]	1998	Case report	Orthodontic extrusion	Enhancement of soft and hard tissue and successful implant placement	4 weeks after extraction	3.75 × 15 mm implants	NR	None	NR
Xi Jiang et al. [15]	2017	RCT (28 patients)	GBR	Good results	Simultaneous implant placement using submerged or transmucosal surgical technique	Dentsply implants	6 months	None	NR
J.P. Sullivan [16]	2013	Case report	Autogenous bone graft from an intraoral site	Good results	3 months after bone grafting	NR	3 years	None	NR
Sezer et al. [24]	2012	Case series (10 patients)	Alveolar Distraction Osteogenesis	Good results	4 months after consolidation of distracted segments	Strauman implants	3 years	In 1 patient a progressive lingual inclination of the distracted segment occurred during distraction. In 1 patient, a transient paresthesia of the area innervated by the inferior alveolar nerve was observed. Infection was observed in 1 case in the postoperative period.	100%
Fagan et al. [17]	2008	Case series (37 patients)	Freeze-dried mineralized bone allograft (FDBA), recombinant human platelet-derived growth factor mixture with a titanium-reinforced membrane and a pediculated connective tissue graft (PCTG) to simultaneously augment hard and soft tissue.	Good esthetic and functional results	Immediate implants (25 patients) Or delayed implants (12 patients)	BioMet. or Nobel Biocare implants	1 year	None	97.3%
Petrungaro et al. [18]	2005	2 Case reports	Allogenic block graft	Good results	Delayed implants	Screw-Vent implants (Zimmer Dental)	1 year	None	NR
Artzi et al. [19]	1997	4 Case reports	Immediate post-extractive implant placement and simultaneous GBR	Good results	Immediate post-extractive implants	Cylindrical implants, Integral Omniloc	2 years	None	NR
Artzi et al. [20]	1997	3 Case reports	GBR and delayed implant placement	Good results	Delayed implant placement, 9 months after GBR	NR	2 years	Early exposure of the e-PTFE membrane in one case, but with no consequences	NR
Crespi et al. [21]	2016	Prospective study (40 patients)	No graft procedure. Spontaneous healing of the extraction socket. Granulation tissue left in situ. Delayed implant placement after 3 months	Good results	Delayed implant placement, 3 months after tooth extraction	Titanium implants with rough surface, Titanium Plasma Spray.	3 months, 3 years	None	100%
Iasella et al. [22]	2003	RCT (24 patients)	Ridge Preservation with freeze-dried bone allograft and a collagen membrane for implant site development compared to extraction alone	Ridge preservation procedures improved ridge height and width dimensions compared to extraction alone.	Delayed implants	Root-form implants	6 months	Some sites showed little dehiscences at the time of implant placement	NR
Barone et al. [23]	2008	RCT (40 patients)	Xenograft for Ridge Preservation after tooth extraction versus Extraction Alone	The ridge preservation procedure significantly limited the resorption of hard tissue after tooth extraction compared to extraction alone.	Delayed implants	NR	7 months	None	NR
Gozneli et al. [27]	2010	Case report	Alveolar Distraction Osteogenesis	Good results	4 months after consolidation of distracted segments, immediate post-extractive implants	Strauman implants	1.5 years	None	NR
Barone et al. [28]	2012	RCT (58 patients)	Spontaneous healing vs. ridge preservation with secondary soft tissue healing	Alveolar ridge preservation technique limits the contour changes after tooth extraction and allows a better preservation of facial keratinized tissue.	Delayed implant placement, 4 months after tooth extraction	NR	8 months	None	NR
Borelli de Barros et al. [29]	2013	Case report	Orthodontic extrusion	Successful implant therapy. Satisfying results	Immediate post-extractive implant	Cone Morse 3.5 × 13 mm. Neodent	6 years	None	NR
Chou et al. [30]	2011	Case report	Orthodontic extrusion	Good esthetic and functional outcomes	Immediate post-extractive implant	5 × 11.5 mm Implant Osseotite, Biomet	2 years	None	NR

**Table 5:** Risk of bias for the included randomized clinical trials (RCT) using Rob 2.0 tool.

Reference	Type of Study	Random Sequence Generation	Allocation Concealment	Selective Reporting	Other Bias	Blinding of Participants and Personnel	Blinding of Outcome Assessment	Incomplete Outcome Data	Conclusion
Xi Jiang et al. [15]	RCT	yes	yes	no	unclear	no	unclear	no	good quality
Iasella et al. [22]	RCT	yes	unclear	no	unclear	no	yes	no	good quality
Barone et al. [23]	RCT	yes	unclear	no	unclear	no	yes	no	good quality
Barone et al. [28]	RCT	yes	unclear	no	unclear	no	yes	no	good quality
Marconcini, Covani et al. [26]	RCT	yes	yes	no	unclear	no	yes	no	good quality

**Table 6:** Risk of bias for the included non-randomized studies using ROBINS-I tool.

Reference	Type of Study	Bias Due to Confounding	Bias in Selection of Participants into the Study	Bias in Classification of Interventions	Bias Due to Deviations from Intended Interventions	Bias due to Missing Data	Bias in Measurement of Outcomes	Bias in the Selection of the Reported Results	Risk of Bias
Amato, Mirabella et al. [11]	Case series	low risk	low risk	low risk	low risk	low risk	moderate risk	low risk	Low risk
Sezer et al. [24]	Case series	low risk	low risk	low risk	low risk	low risk	low risk	low risk	Low risk
Fagan et al. [17]	Case series	low risk	low risk	low risk	low risk	high risk	moderate risk	high risk	Moderate risk
McCarthy et al. [25]	Case series	low risk	low risk	low risk	low risk	high risk	moderate risk	high risk	Moderate risk
Crespi et al. [21]	Prospective study	low risk	low risk	low risk	low risk	high risk	low risk	moderate risk	Moderate risk

**Table 7:** Quality evaluation of the included case reports using CARE guidelines.

Reference	Title (Max 1)	Key Words (Max 1)	Abstract (Max 4)	Introduction (Max 1)	Patient Information (Max 4)	Clinical Findings (Max 1)	Timeline (Max 1)	Diagnostic Assessment (Max 4)	Therapeutic Intervention (Max 3)	Follow-Up and Outcomes (Max 4)	Discussion (Max 4)	Patient Perspective (Max 1)	Informed Consent (Max 1)	Total (Max 30)	Quality of the Study
Joo, Son, Lee [10]	1	0	2	1	3	1	1	4	3	4	4	0	1	25	G
Watanabe et al. [7]	1	0	2	1	2	1	1	2	3	1	3	0	0	18	P
de Barros et al. [29]	1	0	2	1	2	1	1	3	3	2	4	0	0	20	S
Hyun Kim et al. [9]	1	0	3	1	3	1	1	2	3	2	3	0	1	21	S
Chou et al. [30]	1	1	4	1	3	1	1	4	3	1	3	0	0	23	S
Paolone et al. [8]	1	1	2	1	4	1	1	3	3	0	2	0	0	19	P
Holst et al. [13]	1	0	0	1	1	1	1	0	3	1	3	0	0	12	P
Erkut et al. [14]	1	0	1	1	2	1	1	3	3	1	1	0	1	16	P
Mantzikos, Shamus [12]	1	1	2	1	4	1	1	4	3	0	0	0	0	17	P
J.P. Sullivan [16]	1	1	2	1	2	1	1	4	3	0	1	0	0	17	P
Gozneli et al. [27]	1	1	4	1	3	1	1	2	3	3	4	0	1	25	G
Petrungaro et al. [18]	1	1	3	1	1	1	1	0	3	1	1	0	0	14	P
Artzi et al. [19]	1	0	1	1	1	1	1	0	1	1	3	0	0	11	P
Artzi et al. [20]	1	0	1	1	1	1	1	0	1	1	3	0	0	11	P

## Data Availability

Data available upon reasonable request. Review registration: PROSPERO (CRD42020162718).
